# Individualized Theory of Mind (iToM): When Memory Modulates Empathy

**DOI:** 10.3389/fpsyg.2013.00004

**Published:** 2013-02-01

**Authors:** Elisa Ciaramelli, Francesco Bernardi, Morris Moscovitch

**Affiliations:** ^1^Dipartimento di Psicologia and Centro Studi e Ricerche in Neuroscienze Cognitive, Università di BolognaBologna, Italy; ^2^Department of Psychology, University of TorontoToronto, ON, Canada; ^3^Department of Psychology, Northwestern UniversityEvanston, IL, USA; ^4^Rotman Research InstituteToronto, ON, Canada

**Keywords:** theory of mind, empathy, episodic memory, autobiographical memory, episodic simulation

## Abstract

Functional neuroimaging studies have noted that brain regions supporting theory of mind (ToM) overlap remarkably with those underlying episodic memory, suggesting a link between the two processes. The present study shows that memory for others’ past experiences modulates significantly our appraisal of, and reaction to, what is happening to them currently. Participants read the life story of two characters; one had experienced a long series of love-related failures, the other a long series of work-related failures. In a later faux pas recognition task, participants reported more empathy for the character unlucky in love in love-related faux pas scenarios, and for the character unlucky at work in work-related faux pas scenarios. The memory-based modulation of empathy correlated with the number of details remembered from the characters’ life story. These results suggest that individuals use memory for other people’s past experiences to simulate how they feel in similar situations they are currently facing. The integration of ToM and memory processes allows adjusting mental state inferences to fit unique social targets, constructing an individualized ToM.

## Introduction

Humans have the ability and the need to interpret the mental states of other people, including their thoughts, feelings, and intentions. This ability, called “theory of mind” (ToM) is crucial for empathy, which includes representing other people’s thoughts (referred to as “cognitive empathy”) and experiencing affective states aligned to theirs (referred to as “affective empathy”; Amodio and Frith, 2006; de Vignemont and Singer, 2006; Zaki and Ochsner, 2012). Understanding the neural bases and the cognitive mechanisms of ToM is an important goal of research in cognitive neuroscience.

Recent reviews of functional neuroimaging (fMRI) studies have noted that brain regions supporting ToM and cognitive empathy overlap remarkably with those underlying autobiographical memory. The regions of overlap include the medial prefrontal and posterior cingulate cortex, frontal pole, inferior frontal gyrus, regions within the medial temporal lobe, superior temporal sulcus and middle temporal gyrus, and the angular gyrus (Buckner and Carroll, 2007; Rabin et al., 2010; Spreng and Grady, 2010; Zaki and Ochsner, 2012). These regions take part in the “default network,” a set of interconnected brain regions whose activity typically is suppressed by stimulus-driven attention, and enhanced by internally focused activities (Buckner et al., 2008; Spreng and Grady, 2010).

What might be the functional meaning of this overlap? Some authors have suggested that, in order to infer others’ mental states, individuals draw on past experience (Corcoran and Frith, 2003; Gallagher and Frith, 2003; Buckner and Carroll, 2007). Thus, individuals may understand how others feel because they recall having experienced similar episodes personally, and how they felt at that time. Batson et al. (1996), for example, found that participants who had experienced the same electrical shock as their confederates displayed a greater empathic response toward them relative to participants who never had experienced the shock. Likewise, participants who went through a similar experience as the protagonist in a story showed a greater empathic response toward the protagonist relative to participants who had never experienced such an event (Batson et al., 1996). Dimaggio et al. (2008) have shown that the more individuals are able to retrieve and reflect on episodes from their own life narratives, the more likely they are to decipher others’ thoughts and emotions. According to the “episodic simulation hypothesis” (Buckner et al., 2008; Schacter et al., 2008), indeed, one fundamental function of episodic memory, beyond recalling the past, is enabling the formulation of flexible models of the future to inform choice. In the social domain, this may entail retrieving fragments of past events rich in experiential detail, recombining them to construct new scenarios suited to represent the situation currently faced, and pre-experiencing how we, or others, might feel in such situations. The hypothesis of a close relation between episodic memory and ToM processes is supported by evidence that episodic memory and ToM emerge close in time during development (Perner and Ruffman, 1995). Moreover, patient populations with ToM impairments, such as high-functioning autism (Adler et al., 2010) and schizophrenia (Corcoran and Frith, 2003), as well as patients with damage to the ventromedial prefrontal cortex (Stone et al., 1998; Stuss et al., 2001; Shamay-Tsoory et al., 2005, 2009; Ciaramelli et al., 2012) also show a (possibly parallel) impairment in autobiographical recollection (see Gilboa and Moscovitch, 2002; Dimaggio et al., 2012; for reviews), as well as functional abnormalities in the brain default network (e.g., Kennedy et al., 2006; Harrison et al., 2007).

On the other hand, a neuropsychological study of ToM in two amnesic individuals provided contradictory evidence. Rosenbaum et al. (2007) showed that, despite severely impaired autobiographical memory, amnesic patients were normally able to perform standard laboratory ToM tests that required them, for example, to identify whether a character unintentionally said something hurtful to another character, committing what is called a “faux pas” (Faux Pas Test; Stone et al., 1998). The finding that ToM may be intact in amnesic patients with impaired autobiographical memory poses constraints on the purported relation between episodic memory and ToM, indicating that, at least under some circumstances, ToM is independent of episodic memory. As Rosenbaum et al. (2007) speculate, indeed, detection of a faux pas may be achieved through the retrieval of semantic knowledge of how a person might stereotypically feel in a given situation, and of social etiquette (see also Spreng and Mar, 2012). For example, we know that people do not like being told that they look older than they are and we avoid making comments like that. To do so, we do not need to resort to episodic retrieval or simulation. The question remains, therefore, as to whether and why ToM would need the support of episodic simulation processes.

As much as semantic memory is about scripts and general knowledge about the world, it is not suited to capture the characteristics of unique individuals and unique situations. Exclusive use of semantic representations may be sufficient to infer what the average person is likely to experience in a given situation, which is what is required by most standardized ToM tests that employ strangers as the social targets (e.g., Stone et al., 1998; Baron-Cohen et al., 2001; Rabin et al., 2010; Spreng and Grady, 2010; St. Jacques et al., 2011). However, we typically do not reason about average strangers, but about distinct individuals, some of whom we know well, and with whom we interact. In this case, semantic representations, which only allow for stereotyped interpretations of others’ behavior, are likely to be insufficient to make adaptive mental state inferences. In this case, episodic memory for shared experience may be necessary to tailor ToM processes on the social target we are interpreting, to construct what we call an “individualized” ToM (iToM). Suppose, for example, you have a friend that for all of his life has been said to look young for his age, and this was a problem in trying to get positions of responsibility. In this situation, one would abandon (semantic) social etiquette, and create instead a new, *ad hoc* rule of avoiding commenting on his youthful appearance.

### The present study

Our hypothesis is that episodic memory is necessary to retrieve previously acquired information in order to derive tailored interpretations of others’ behavior, allowing one to adjust ToM to fit unique social targets (iToM). To test this hypothesis, in the present study we had participants read about the lives of two individuals, one extremely unlucky in love, whose life was punctuated with episodes involving failures in intimate and romantic relationships, and the other extremely unlucky in professional life, whose life was punctuated with failures at school and at work. The two life narratives were rich in episodic and experiential detail. Participants’ immediate free recall of the narratives provided us with a measure of recollection. Next, participants considered social scenarios that could or could not contain a faux pas, that is, a situation in which a character unintentionally hurt a second character (the victim), and participants had to detect the faux pas and report how much empathy they felt for the victim. We assessed both cognitive empathy and affective empathy. Critically, in some scenarios the faux pas concerned the victim’s intimate/romantic relationships (Love scenarios), whereas in other scenarios it was about the victim’s professional relationships (Work scenarios). For comparison purposes, generic violations of social norms not specifically involving intimate/romantic or professional relationships were also used. The type of social violation and the identity of the victim were experimentally crossed, such that the victim in the scenarios could be the characters participants had read about – one unlucky at work, one unlucky in love – as well as another character about whom participants knew nothing.

Our main prediction was that empathy toward the victim of a faux pas should be modulated by memory for the victim’s life story. Thus, we expected more empathy for the victim unlucky in love in Love scenarios, and for the victim unlucky at work in Work scenarios. If this memory-driven modulation of empathy is based on the recollection of the victim’s story, then a correlation should be expected between the entity of the modulation and the amount of detail recalled from victim’s life story. We also investigated whether memory for the victim’s life story would predict a better ability to detect social violations, i.e., ToM accuracy. To the extent that episodic memory and ToM recruit overlapping neural networks (Buckner and Carroll, 2007), one would expect a correlation between recall accuracy and faux pas recognition accuracy. On the other hand, it has been reported that trait empathy may be related to ToM and social cognitive functioning (Zaki and Ochsner, 2012). For example, individual differences in self-report measures of empathy track with activity in brain regions associated with mentalizing and ToM (Wagner et al., 2011). Moreover, patients with lesions in the ventromedial prefrontal cortex exhibit both low self-reported cognitive empathy (Shamay-Tsoory et al., 2009) and impaired understanding of other people’s thoughts and intentions (Stone et al., 1998; Shamay-Tsoory et al., 2005; Ciaramelli et al., 2012). On this view, one may expect that participants with high levels of trait empathy, especially cognitive empathy, would be better able to adopt the perspective of the victims in the scenarios, and therefore adjust their empathic response depending on the identity of the victim.

## Materials and Methods

### Participants

Thirty-one individuals were recruited for the study. Participants were tested at the Department of Psychology of the University of Toronto, Canada, and at the Department of Psychology of Northwestern University, USA. Two subjects were excluded from the study because part of the data was lost as a result of technical problems. The experimental group, therefore, was composed of 29 participants (11 males), with mean age of 22.38 years (range 18–37) and a mean education of 15.03 years (range 13–19). For four of the participants, cognitive empathy scores were not registered due to a malfunctioning of the computer program. Therefore, all the analyses involving cognitive empathy were run on 25 of the 29 participants. Participants were not taking psychoactive drugs, and were free from current or past psychiatric or neurological illness as determined by history. Participants gave written informed consent for the study, which was approved by the ethics committees of the University of Toronto and of Northwestern University. They received course credit or $10.

### Materials

The materials included two “life-stories” narrating excerpts from the lives of two fictitious characters, a faux pas recognition task, and the Interpersonal Reactivity Index Questionnaire (IRI; Davis, 1980).

#### Life-stories recall task

We created two stories narrating the lives of two fictitious characters, in the form of a series of episodes narrated in first person (see Life-stories in Appendix). The life story of one character concerned a series of events mainly involving failures in intimate and romantic relationships. For male participants in the experiment, this character, unlucky in love, was called Mike, whereas for female participants the character was called Susan. Life-stories were slightly adapted depending on the gender of the protagonist (see Life-stories in Appendix). The life story of the other character, on the other hand, concerned a series of events mainly involving failures in professional life. This character, unlucky at work, was called Adam and Jean for male and female participants, respectively. Thus, while Mike/Susan’s life was punctuated by unsuccessful love-related events (e.g., *I just asked her for her name and she answered: “I am sorry, I am very busy and cannot waste my time!*”), Adam/Jean’s life mainly involved work-related failures (e.g., *I have been hired recently, but I already had a hard time with my boss. He said that I had mistakenly filed some invoices, and that was a disaster*). Life-stories were rich in spatial, perceptual, and emotional details (e.g., […] *the flat is on Queen West, on the second floor of a Victorian house painted in red with a blue ceiling It’s just that sometimes I can feel like such a nothing*). Life-stories were subdivided *a priori* in conceptual units, each of which conveyed a bit of information, such as a unique occurrence, fact, statement, thought, etc. (see also Levine et al., 2002 for a similar segmentation procedure). In the first version of the life-stories, which was administered to participants tested at University of Toronto (*N* = 14), the love-related story contained 199 conceptual units, and the work-related story contained 233 conceptual units. Participants subsequently tested at Northwestern University (*N* = 15) received a slightly shortened version of the love-related story and the work-related story, which both contained 171 conceptual units.

#### Faux pas recognition task

Thirty-seven social scenarios were selected and adapted from the “Faux Pas Recognition Test” (Baron-Cohen et al., 1999) or the “Social Stories Questionnaire” (Lawson et al., 2004), or created *ad hoc* (see Faux Pas Scenarios in Appendix for the list of scenarios used). Twenty-seven scenarios contained a violation of accepted social norms (a faux pas): an individual said something awkward that may hurt someone (the victim). The remaining 10 scenarios involved neutral social interactions that did not contain any faux pas (Neutral scenarios). In eight scenarios, the faux pas hit the victim on his/her intimate/romantic relationships (Love scenarios; e.g., […] *Susan wondered whether her boyfriend also remembered their anniversary. Then he came out of the shower. “What were you saying honey? I couldn’t hear from the shower,” asked the boy. Susan said: “I thought it would be nice to go out for dinner since today…” But he interrupted her: “Oh sorry honey, not today: I promised some colleagues I would join them for a drink! What about next Saturday?”*). In nine scenarios, the faux pas was about the victim’s professional life (Work scenarios; e.g., *Susan went to say bye to the director of her office, because she was planning to leave for her holidays. [*…*]*. “*Don’t worry,” answered the director, “I don’t think your absence will cause huge problems. Have fun!*”). Ten scenarios involved generic social violations that did not pertain specifically to the victim’s intimate/romantic or professional life (Generic scenarios; e.g., *Susan had just met an Italian colleague, Paola. [*…*] Susan wanted to invite Paola for dinner to get to know her better*. “*Hey Paola, why don’t you come over for dinner some time, we just bought a pasta machine and you could help us figure how to use it!” Paola answered: “Oh why not… but I guess you should be able to get it to work. Wasn’t there an instruction manual?*”).

All participants considered 20 scenarios: five Love scenarios, five Work scenarios, five Social scenarios, and five Neutral scenarios. Participants received slightly different sets of scenarios. This was done as part of a pilot research project testing the generalization of our effects to different types of materials, which was later discontinued. In all our analyses, we assessed (and controlled for) the effect of the different set of scenarios used. Twenty participants received Set A (Love scenarios #: 1, 2, 3, 4, 5; Work scenarios #: 1, 2, 3, 4, 5; Generic scenarios #: 1, 2, 3, 4, 5; Neutral scenarios #: 1, 2, 3, 4, 5; see Faux pas scenarios in Appendix), 4 participants received Set B (Love scenarios #: 1, 4, 6, 7, 8; Work scenarios #: 4, 6, 7, 8, 9; Generic scenarios #: 6, 7, 8, 9, 10; Neutral scenarios #: 3, 4, 6, 7, 8), and five participants received Set C (Love scenarios #: 1, 4, 6, 7, 8; Work scenarios #: 4, 6, 7, 8, 9; Generic scenarios #: 6, 7, 8, 9, 10; Neutral scenarios #: 4, 6, 8, 9, 10). Each scenario was presented three times. One time the victim of the faux pas was Mike/Susan (the character unlucky in love), one time it was Adam/Jean (the character unlucky at work), and one time it was Jason/Patricia, a character participants did not know anything about. The order of the scenarios was randomized for each participant.

The participants’ task was to consider each scenario, and judge whether it did or did not contain a faux pas by pressing one of two buttons. For affirmative responses, participants additionally answered two questions regarding the amount of cognitive empathy (i.e., “How bad do you think [victim] felt from one (not bad) to seven (very bad)?”) and affective empathy (i.e., “How bad do you feel for [victim] from one (not bad) to seven (very bad)?”) they felt for the victim.

#### Interpersonal reactivity index

The IRI is a self-report scale assessing four main aspects of empathy. The “perspective taking” (PT) scale assesses the ability to adopt spontaneously the perspective of other people; the “fantasy” (F) scale assesses the tendency to identify with characters in movies, novels, and other fictional situations; the “empathic concern” (EC) scale assesses the tendency to experience feelings of warmth, compassion, and concern for others undergoing negative experiences; the “personal distress” (PD) scale assesses feelings of anxiety and distress for people going through sufferings and afflictions (Davis, 1980).

### Procedure

Each participant was scheduled for an individual experimental session. First, participants were asked to read the two life-stories and were informed that, after reading each story, they would have to recall it in as much detail as they could. The order of presentation of the two life-stories was counterbalanced across participants. There was no time limit to read and recall the life-stories, which took about 10 min in all cases. Participants’ reports were recorded. All data were collected and scored by author FB. Scoring involved counting the number of conceptual units correctly recalled, a procedure similar to the one used for the Logical Memory subtest of the Wechsler Memory Scale (Wechsler, 1987). At the time of scoring, FB was blind to the results participants had attained in the faux pas recognition task. An additional rater, blind to the experimental hypotheses, rated 50% of the recall reports. Inter-rater agreement was high (*r* = 0.96).

Participants then underwent the faux pas recognition task. They were told that the task consisted in reading social scenarios, and deciding, for each scenario, whether it contained a faux pas, that is, a situation in which someone said something inconvenient or awkward that may hurt another person, and how they felt for the victim. Participants were encouraged to “put themselves in the victim’s shoes” while considering the scenarios. They were also informed that the scenarios may involve the characters they had read about previously. After completing the faux pas recognition task, participants completed the IRI questionnaire.

## Results

### Faux pas recognition accuracy

Table 1 reports participants’ accuracy in detecting faux pas, by type of scenario and character. An analysis of variance (ANOVA) on accuracy scores with Scenario (Love scenarios, Work scenarios, Generic scenarios, and Neutral scenarios) and Character (unlucky in love, unlucky at work, unknown) as within-subject factors, and Set (A, B, C) as between-subject factor, revealed a significant effect of Scenario [*F*(3,78) = 3.24; *p* < 0.05, $\eta_{p}^{2}=0.11$]. *Post hoc* Duncan comparisons showed that subjects attained a lower recognition accuracy in detecting work-related faux pas compared to love-related faux pas, generic faux pas, or the absence of faux pas (Neutral scenarios; *p* < 0.05 in all cases). The effect of Set was significant [*F*(2,26) = 4.32; *p* < 0.05,$\eta_{p}^{2}=0.24$], such that a lower recognition accuracy was associated to Set A compared to Set C scenarios (0.79 vs. 0.94; *p* < 0.05). The factor Set, however, did not interact in a significant way with any of the other variables (*p* > 0.47, $\eta_{p}^{2}<0.06$) in all cases. The Scenario × Character interaction was not significant (*p* = 0.79, $\eta_{p}^{2}=0.01$), indicating that the identity of the victim did not influence participants’ ability to detect faux pas in social interactions (or the lack thereof).

**Table 1:** **Mean faux pas recognition accuracy by type of scenario and character**.

	Love scenarios	Work scenarios	Generic scenarios	Neutral scenarios
Unlucky in love	0.90 (0.03)	0.70 (0.04)	0.81 (0.04)	0.90 (0.02)
Unlucky at work	0.91 (0.02)	0.75 (0.04)	0.80 (0.04)	0.91 (0.03)
Unknown	0.88 (0.03)	0.69 (0.04)	0.80 (0.04)	0.90 (0.03)

*Values in parentheses represent the standard error of the mean*.

### Cognitive and affective empathy

**Figure 1:**
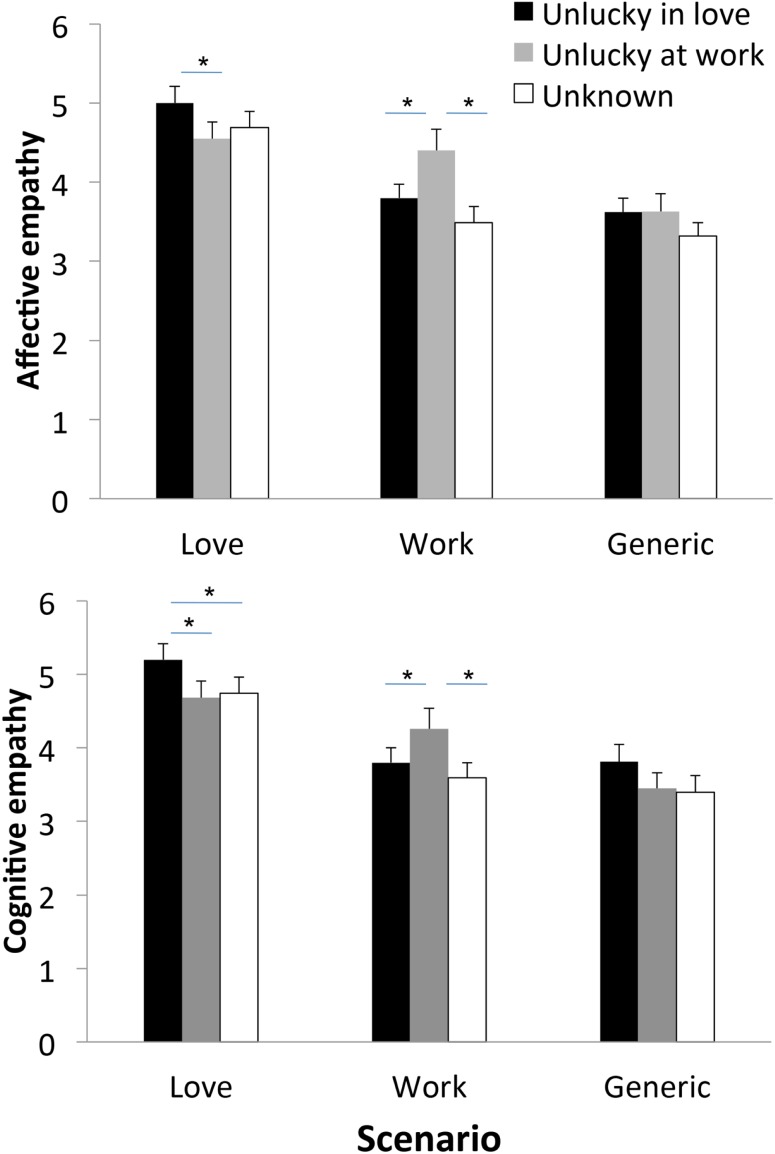
**Mean affective empathy scores (TOP panel) and cognitive empathy scores (BOTTOM panel) by type of scenario and character**. Error bars indicate the standard error of the mean. *p < 0.05.

We next investigated whether individuals’ empathy toward the victim of a faux pas depended on the identity (and story) of the victim. We first analyzed affective empathy scores. An ANOVA on affective empathy scores with Scenario (Love, Work, Generic), Character, and Set as factors revealed a significant effect of Scenario [*F*(2,52) = 15.71; *p* < 0.0001, $\eta_{p}^{2}=0.37$], and a significant effect of Character [*F*(2,52) = 5.41; *p* < 0.01,$\eta_{p}^{2}=0.17$], which were qualified by a significant Scenario × Character interaction [*F*(4,104) = 6.40; *p* < 0.0001,$\eta_{p}^{2}=0.19$]. *Post hoc* Duncan comparisons showed that affective empathy scores in Work scenarios were higher for the character unlucky at work than for the character unlucky in love (*p* < 0.001) or the unknown character (*p* < 0.0005), with no difference between the character unlucky at work and the unknown character (*p* = 0.10). Conversely, affective empathy scores in Love scenarios were higher for the character unlucky in love than for the character unlucky at work (*p* < 0.05), and, marginally, for the unknown character (*p* = 0.07), with no difference between the character unlucky at work and the unknown character (*p* = 0.41). In contrast, affective empathy scores were not modulated by the character’s identity in Generic scenarios (*p* > 0.09 in all cases; see Figure 1). The factor Set was not significant, and did not interact in a significant way with any of the other variables (*p* > 0.61, $\eta_{p}^{2}<0.05$ in all cases).

Conceptually similar results were obtained on cognitive empathy scores. The ANOVA on cognitive empathy scores evinced a significant effect of Scenario [*F*(2,44) = 12.14; *p* < 0.0001, $\eta_{p}^{2}=0.35$], and a marginal effect of Character [*F*(2,44) = 3.02; *p* = 0.058, $\eta_{p}^{2}=0.12$ ]. The Scenario × Character interaction just failed to reach conventional levels of statistical significance [*F*(4,88) = 2.45; *p* = 0.051, $\eta_{p}^{2}=0.10$], likely due to the fact that this analysis was run on 25 subjects only. Because the effect size for the Scenario × Character interaction is in the medium range ($\eta_{p}^{2}=0.10$), and for completeness, we ran *post hoc* Duncan comparisons. Cognitive empathy scores in Work scenarios were higher for the character unlucky at work than for the character unlucky in love (*p* < 0.05) or the unknown character (*p* < 0.01), with no difference between the latter two (*p* = 0.29). Conversely, cognitive empathy scores in Love scenarios were higher for the character unlucky in love than for the character unlucky at work or the unknown character (*p* < 0.05 in both cases), with no difference between the latter two (*p* = 0.76). In contrast, cognitive empathy scores were not modulated by the character’s identity in Generic scenarios (*p* > 0.06 in all cases; see Figure 1). Again, the factor Set was not significant, and did not interact in a significant way with any of the other variables (*p* > 0.53, $\eta_{p}^{2}<0.06$ in all cases).

These findings indicate that empathic responses are influenced by the victim’s identity and type of social violation s/he is facing, consistent with the hypothesis that retrieval of past episodes about others influences our appraisal of similar social situations involving the same individuals. This hypothesis is explored further in the next section.

### Empathy modulation, episodic memory, and empathy scales

We next investigated whether the degree to which participants modulated their empathic responses depending on the victim’s identity correlated with their ability to recall the victim’s story in detail, and to standard self-report measures of empathy as assessed in the four subscales of the IRI (F, PT, EC, PD).

We calculated a recall accuracy score as the number of conceptual units recalled out of the total number of conceptual units (collapsed across the two life-stories). Recall accuracy was highly variable across participants, ranging from 0.07 for individuals who merely reported the gist of the stories to 0.36 for individuals who reported numerous vivid qualitative details (Mean = 0.21; SD = 0.08). We also calculated an index of memory-based empathy modulation. For Love scenarios, the index was calculated as (Empathy_unlucky in love_ − Empathy_unlucky at work_)/Empathy_unknown_, and it indicated the difference between the amount of empathy felt in Love scenarios toward the victim unlucky in love minus that toward the victim unlucky at work, adjusted for the participant’s general tendency to empathize, i.e., empathy toward the unknown victim. The same index was calculated for empathy scores in Work scenarios as (Empathy_unlucky at work_ − Empathy_unlucky in love_)/Empathy_unknown_. The two indices were summed to get a general empathy modulation index. The empathy modulation index was calculated for cognitive empathy scores and affective empathy scores separately.

**Figure 2:**
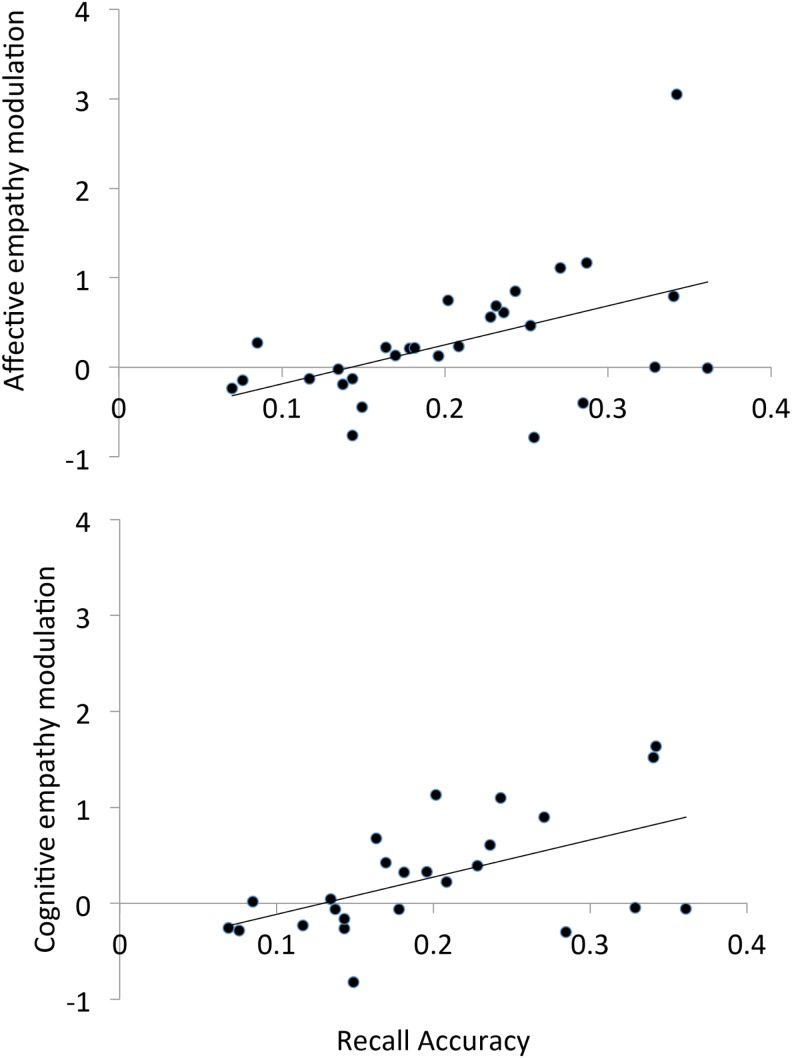
**Scatter plot of the bivariate correlation between recall accuracy for the life-stories and the affective empathy modulation index (TOP panel) and the cognitive empathy modulation index (BOTTOM panel)**.

In order to investigate the relation between the empathy modulation indices and recall accuracy and self-report measures of empathy, while controlling for the effect of Set, we ran an Analysis of Covariance (ANCOVA) on affective and cognitive empathy modulation scores, with Set as between-subject factor, and recall accuracy, and the scores at the F, PT, EC, and PD subscales of the IRI as covariates. The ANCOVA on affective empathy modulation scores showed that the affective empathy modulation index correlated with recall accuracy [β = 0.70, *F*(1,21) = 8.69, *p* < 0.01, $\eta_{p}^{2}=0.29$; see Figure 2], but not with the scores at the IRI subscales (*p* < 0.52, $\eta_{p}^{2}<0.02$ in all cases). There was no significant effect of Set (*p* = 0.27, $\eta_{p}^{2}=0.11$). The same ANCOVA on cognitive empathy modulation scores showed that the cognitive empathy modulation index correlated positively with recall accuracy [β = 0.79, *F*(1,17) = 10.69, *p* < 0.005, $\eta_{p}^{2}=0.38$] (see Figure 2), but not with the scores at the IRI subscales (*p* > 0.37, $\eta_{p}^{2}<0.05$ in all cases). There was no significant effect of Set (*p* = 0.14, $\eta_{p}^{2}=0.20$).

### Faux pas recognition accuracy, episodic memory, and empathy scales

Finally, we investigated whether the ability to detect faux pas was related to recall accuracy for the victims’ life-stories, and to standard self-report measures of empathy. An ANCOVA on faux pas recognition accuracy with Set as between-subject factor, and recall accuracy and the scores at the F, PT, EC, and PD subscales of the IRI as covariates, yielded no significant effect of recall accuracy (*p* = 0.52; $\eta_{p}^{2}=0.02$), IRI scores (*p* > 0.44; $\eta_{p}^{2}<0.03$ in all cases), or Set (*p* = 0.14; $\eta_{p}^{2}=0.16$). Similar results were obtained if we focused on recognition accuracy for Work scenarios and Love scenarios, whose contents are related to the main themes of the victims’ life-stories, and recognition accuracy for Generic and Neutral scenarios, whose contents are not related to memory contents (*p* > 0.07, $\eta_{p}^{2}<0.20$ in all cases).

## Discussion

Understanding other people’s thoughts and feelings in a given situation is adaptive if we have a distinctive representation of who they are, and how they have behaved in similar situations. This enables us to anticipate their reactions to the current situation as accurately as possible. The present study shows that episodic memory for others’ past experiences modulates significantly our appraisal of, and reaction to, what is happening to them currently.

Participants read the life-stories of two characters; one had experienced a long series of love-related failures, the other a long series of work-related failures. Consistent with the hypotheses, in a later faux pas recognition task, they reported more empathy for the character unlucky in love if she or he was the victim of an additional love-related faux pas than they did for the character unlucky at work or for an unknown character. Analogously, more empathy was reported for the character unlucky at work receiving yet another work-related social violation than it was reported for the character unlucky in love or an unknown character. It is worth emphasizing that participants did not appear to be generally more empathic toward the two familiar characters compared to the unknown character (e.g., Stinson and Ickes, 1992). The increase in empathy for the familiar characters, indeed, was highly situation-specific: participants felt more empathy toward the character unlucky in love in love-related scenarios, but treated this character normally in work-related scenarios, and vice versa for the character unlucky at work. Additionally, all characters attained comparable levels of empathy in Generic scenarios, whose contents were not relevant to the life story of either character. Rather, the present results suggest that while contemplating faux pas scenarios, individuals recollected previous episodes involving the victim analogous in content to the situation she currently faces, and how the victim had felt in those situations. Retrieval of past episodes involving the victim allowed a better simulation of how she felt in response to the current faux pas, or, in other words, the construction of an iToM, shaping individuals’ empathic responses accordingly.

The present findings are in line with the “episodic simulation hypothesis” (Schacter et al., 2008; see also Buckner and Carroll, 2007), according to which retrieval of past experiences is needed to envisage fictitious events, and make decisions based on such simulations. In line with this, the amount of detail recalled from the victims’ life-stories correlated positively with the degree to which individuals modulated their empathic responses depending of the identity of the victim, suggesting that a greater availability of episodic memories from the victims’ life helped them to envisage their feelings in similar situations more vividly and faithfully (see also Spreng and Mar, 2012, for a Discussion). These results make contact with findings from a recent fMRI study (Perry et al., 2011), in which hippocampal activity was detected while subjects made emotional judgments about people deemed similar to themselves and facing events that had occurred in their own life (e.g., How would Joe feel about losing his wallet?), suggesting that individuals may resort to personal memories in order to understand other people better and empathize with them. Of course, different from Perry et al.’s (2011) paradigm, in ours subjects did not need to use the self (and personal memories) as a proxy to empathize with the victims (Mitchell et al., 2006), as they had the original memories of the victims’ past experiences available.

Our findings fit nicely with the results of a recent fMRI study investigating the relation between autobiographical memory and ToM (i.e., imagining the thoughts and feelings of another person) for personally known versus unfamiliar others (Rabin and Rosenbaum, 2012). It was found that brain regions supporting ToM for personally known others overlapped more closely with those supporting autobiographical memory than did regions supporting ToM for unknown others, and the overlap was maximal in midline regions, including the hippocampus. This finding suggests that in order to imagine the mental states of known people individuals rely, to some extent, on shared past experience (Rabin and Rosenbaum, 2012; see also Rabin et al., 2012b). Consistently, a previous fMRI study had shown that the medial prefrontal cortex responded more strongly when participants made judgments about the personal preferences of friends and close others, which arguably are likely to trigger relevant past experiences, relative to strangers, regardless of whether the other person is perceived as similar to oneself (Krienen et al., 2010). On the other hand, ToM for unfamiliar others was associated with activity in more lateral frontal and temporal regions associated with accessing semantic knowledge (e.g., Martin and Chao, 2001), consistent with the idea that to infer strangers’ mental states reliance on social scripts and general knowledge may be sufficient (Rosenbaum et al., 2007).

Previous research has shown that episodic memory retrieval is related to simulation of future events, and functional to social cognition. Amnesic patients with problems at remembering specific episodes from the past, indeed, may also exhibit problems at imagining specific episodes in the future (Tulving, 1985; Rosenbaum et al., 2005; Race et al., 2011; but see Squire et al., 2010), and their constructions of fictitious events appears significantly reduced in richness and content compared with those of controls (Hassabis et al., 2007). Crucially, it has been demonstrated that problems in simulating fictitious events has an impact on social problem solving. Sheldon et al. (2011) had patients with unilateral temporal lobe epilepsy and excisions (TLE), older adults, and control participants describe detailed solutions to various open-ended, social scenarios. TLE patients and older adults, both having deficits in episodic memory, provided fewer steps relevant to the given solution than their comparison group, and their descriptions of the step was made of fewer internal (episodic) details but a similar number of external (semantic) details compared to their control groups. Thus, even though amnesic patients can solve standardized ToM tasks normally (Rosenbaum et al., 2007), memory problems may in fact hinder performance in ill-defined, real world social situation that require evaluating the outcome of multiple, alternative mental simulations of the situation being considered, and integrating context-specific with person-specific information, tapping episodic simulation processes to a greater extent. Consistent with this interpretation, Levine et al. (1998) described a patient, ML, who had sustained damage to the right uncinate fasciculus, a band of fibers which connects the medial temporal lobe with ventral frontal cortex, and who had severe autobiographical memory deficits associated with problems in real world social interactions. Interestingly, ML had difficulty knowing how to behave around family members and friends and, despite being able to re-learn socially acceptable behavior under structured routines, he remained unable to self-regulate his behavior in unstructured situations (Levine et al., 1998).

It has been noted that both re-experiencing the past and inferring others’ mental states require the ability to consider alternatives to events in the immediate environment, or self-projection (Buckner and Carroll, 2007; Mitchell, 2009), be this toward another time (for episodic memory) or another person’s perspective (for ToM). One way to interpret the overlap in brain activity between episodic memory and ToM, therefore, is as structural in nature: both activities are supported by the same neural circuitry, the one that enables self-projection. Were the relation between episodic memory and ToM merely structural, however, one would expect a correlation between episodic memory and ToM performance. However, in the present study free recall (of the life-stories) was not related to faux pas recognition accuracy, and this held even if we focused on Love and Work scenarios, whose contents resonated with memory contents. This result is compatible with previous evidence showing that patients with significant episodic memory problems can attain normal accuracy in ToM tasks, including faux pas recognition tasks (Rosenbaum et al., 2007; Rabin et al., 2012a). Additionally, faux pas recognition accuracy was not related to “PT” scores in the IRI, as the self-projection hypothesis would predict. Our results, therefore, are more consistent with the view that ToM systems, though inherently sufficient to decipher social situation/violations, may co-opt episodic memory systems to integrate flexibly the characteristics of the situation with those of the victim, modulating empathic responses accordingly. This suggests a functional relation between episodic memory and ToM that is more in line with the episodic simulation hypothesis.

The “functional” (as opposed to “structural”) interpretation proposed is also in line with the fact that we found largely parallel effect of episodic memory on cognitive empathy and affective empathy, while only the brain regions supporting cognitive empathy overlap with those supporting autobiographical memory (de Waal, 2008; Shamay-Tsoory et al., 2009; Zaki and Ochsner, 2012). In contrast, affective empathy is related to the ability to share others’ emotional experiences through mirroring neural mechanisms (Preston and de Waal, 2002; Gallese et al., 2004; Singer and Lamm, 2009). Note, however, that mirroring occurs (and has been investigated) typically when perceivers make use of observable cues about what another person is feeling, whereas self-projection is mostly engaged when inferring the mental states of individuals that are not physically present (Zaki and Ochsner, 2012). Because in the present study participants made both cognitive and affective empathy judgments for individuals who were removed from their current experience, both judgments likely relied on, and were modulated by, the same type of (memory) cues (see de Vignemont and Singer, 2006, for other evidence for the contextual modulation of affective empathy). Indeed, the cognitive and the affective modulation indices were highly correlated in our sample (*r* = 0.83). An additional reason why cognitive empathy and affective empathy may have been aligned in our study is that participants were young individuals, likely struggling with similar love- and work-related issues as the protagonists in the two stories. Thus, while reading the faux pas stories, participants may not only have inferred what the characters unlucky in love and the character unlucky at work felt, but also shared their feelings because, to some extent, the saw bits of their own life in the lives of the fictitious characters. Future studies should investigate whether the degree to which memory for others’ life resonates with one’s own biography modulates the relation between cognitive and affective empathy (see also Batson et al., 1996).

A number of alternative interpretations to our data deserve consideration. Since in the present study recall accuracy was not experimentally manipulated, it is possible that a third variable, for example an initial empathic response while reading the life-stories, may have influenced both subsequent recall and empathic responses to social violations. In support to this hypothesis, recall accuracy correlated positively, though not significantly, with empathy scales (*F* scale: *r* = 0.35; *p* = 0.056; PD scale: *r* = 0.36; *p* = 0.051). However, the fact that the memory-based empathy modulation correlated with recall accuracy but not with measures of empathy suggests that it was episodic memory, not empathy, that drove the *situation-specific* adjustments in empathy for known individuals.

Another possibility is that, instead of episodic memory, a semantic labeling of the characters as “the unlucky in love” and “the unlucky at work,” or implicit emotional associations (see Lieberman et al., 2001) supported situation-specific empathic responses in the current study. Lieberman et al. (2001), for example, have shown that, in a choice paradigm, amnesic patients show a normal tendency to revise their attitudes to fit a counter-attitudinal behavior, in the absence of explicit memory for that behavior. Although we cannot exclude that semantic or implicit memory contributed to our results, the fact that the modulation of empathic responses tracked the amount of detail in participants’ recollection makes it unlikely that it derived merely from semantic or implicit memory.

This study has a number of limitations. First, our conclusions need to be confirmed with different materials. As Stone et al. (1998) noted, detecting a faux pas requires two things: (1) understanding that one person has knowledge that the other person is unaware of, or a mistaken belief, and (2) the empathic understanding of what kind of things someone (the victim) would find upsetting. In our experimental paradigm, episodic memory had an impact on this latter factor, tuning participants to the victims’ inner motives. One may expect, then, that if the faux pas is subtle, or it depends relatively more on the victim’s idiosyncrasies (point #2) rather then on “cold” aspects of ToM (point #1), then an impairment in episodic memory may prevent one from detecting a faux pas in the first place, having an impact on faux pas recognition accuracy. In the extreme case of the example we made in the Introduction, one would not call telling someone that he looks tremendously young for his age a faux pas without having memory for his life. Thus, future studies using more subtle social scenarios that cannot be deciphered completely within ToM systems or through abstract social knowledge would be important to test the relation between episodic memory and ToM accuracy further.

Moreover, it should be noted that the present results are limited to the healthy population we tested. In clinical populations (e.g., autistic patients, schizophrenic patients, patients with personality disorders), impairments in autobiographical memory, and ToM may co-occur and be related to each other (Corcoran and Frith, 2003; Adler et al., 2010; see Dimaggio et al., 2012 for a review). Interestingly, some therapeutic approaches for personality disorders and schizophrenia (e.g., Lysaker et al., 2007, 2011; Dimaggio and Attinà, 2012; Dimaggio et al., 2012) insist on the importance of eliciting patients’ specific memories of relevant social interactions (as opposed to resorting to overgeneralized memories), to help patients appreciate psychological causalities, and track down the mental states of the individuals involved more accurately. For example, Dimaggio and Attinà (2012) described a patient with a narcissistic personality disorder who arrived demoralized at one session reporting that he had been socially rejected by two peers at a party. By re-exploring the original event with the therapist, he was able to recall additional contextual details (“There were more of us, and we were more familiar with each other. And to tell the truth, we weren’t paying them much attention”), and consider the alternative possibility that his peers ignored him because they felt uncomfortable at the party, not because something was wrong with him (Dimaggio and Attinà, 2012, p. 931). The fact that, in clinical populations, the retrieval of past episodes promotes the explicit consideration of others’ mental states is in line with the results of the present study. One question for future research is whether such memory-driven improvements in ToM depend on accessing specific contents about past episodes, or on the repeated activation of the neural network that supports both episodic memory and cognitive empathy.

Finally, we believe that an important step toward specifying the relation between autobiographical memory and ToM, would be to adopt both subjective and objective measures of ToM (see Zaki et al., 2008, for a measure of empathic accuracy). For example, most studies, including the present study, require participants to explicitly consider the mental states of other people, but do not assess whether participants did infer these mental states correctly (Rabin et al., 2010; Spreng and Grady, 2010; Rabin and Rosenbaum, 2012). As well, self-report measures of empathy may not track with the actual ability to read, and resonate with, others’ mental states, especially in clinical populations (see Ritter et al., 2011; Dimaggio et al., 2012). This factor may also explain the lack of correlation between self-report empathy and ToM performance in the present experiment. Investigating the relation between episodic memory, and subjective as well as objective measure of ToM will reveal whether there is a causal relation between the two processes, or whether they represent two instances, not necessarily intertwined, of imagining a perspective removed from current experience.

To conclude, the present study shows that the retrieval of memories of previous episodes influences participants’ current social processing significantly, such that empathy toward the victim of a social violation is modulated by memories involving the victim that bear a resemblance to the situation she or he is currently facing. These findings suggest that understanding others’ thoughts and feelings entails integrating flexibly information about past experience and more contingent information, constructing detailed simulations of social targets and situations that preserve their uniqueness, a function we call individualized ToM.

## Conflict of Interest Statement

The authors declare that the research was conducted in the absence of any commercial or financial relationships that could be construed as a potential conflict of interest.

## Appendix

### Life-stories

#### Mike/*Susan* (unlucky in love; *Toronto version)*

My name is Mike/*Susan*|| and I am 30 years old.|| I was born in Dublin,|| but now I live in downtown Toronto||, in my own flat||. The flat is on Queen West,|| on the second floor|| of a Victorian house|| that is painted in red|| and with a blue ceiling.|| I still remember the moment I decided to rent this flat.|| I was wandering in Queen West when I bumped into this enchanted house for rent||. The landlord was there||, a funny|| Portuguese guy||. We talked for a while and the deal was done!|| I am so happy to live on Queen West||. I feel relieved that I can walk in my neighborhood||. It’s like a breath of fresh air to me.|| Yet, I’m very close to my family|| and sometimes it’s tough to be apart||. I spent such a pleasant childhood|| in Dublin||, mainly because we lived on a farm.|| I can still smell the cookies|| that my granny used to bake|| on Sunday mornings.||

I have always known I would leave, though||. When I was in sixth grade||, I got an award from my school|| for the best essay|| about organ donation||. I remember I was so proud of myself||. Even though I was really young, I was able to explain the pros and cons about organ donation.|| For example, I wrote about two parents that had lost their child||. They didn’t want to donate their child’s organs||, but the doctor told them that they would save|| three lives and||, somehow, their child would keep on living.|| So they allowed the donation||. I believe it was very simple for the readers to guess what my personal take on the topic was:|| I truly agree with people who accept to donate their organs,|| and I would do that myself.|| Indeed, even though I have little spare time,|| I still keep on promoting the cause for donation||. Also, I never forget about giving blood.|| Since I was very young, I have always been interested in the field of health.||

After graduating in Pharmacy|| at the University of Toronto,|| I found a job in the Pharmaceutical industry||. The name of the industry is “Atom”||, it is located downtown, between Dundas and Manning.|| You might have noticed the place, it is a colored building|| and there is a dog at the entrance||, Zac||, a very cute beagle|| that we all love||. I am fairly happy with the job|| and I especially enjoy working with my colleagues.|| They are fun,|| of my age,|| and collaborative||. I feel good there|| and it is important because, apart from them, I do not have many friends here in Toronto.||

I mean, I have a very small group of close friends||. We enjoy drinking beer while watching baseball games or planning trips/*We enjoy drinking coffee while watching TV or planning trips*||. Sometimes we go to our favorite club to hit on girls/*boys*||. Our favourite club is “Coca”||. It is on Queen street West||, very close to my place.|| One of my closest friends is Simon/*Kate*.|| He’s a very good looking guy/*She’s very pretty*,|| and, when we all hang out together, he always gets the cutest girls/*boys*.|| Simon/*Kate* doesn’t need to try very hard to attract girls/*boys*||. He/*she* just starts talking about random topics and they listen to him/*her*||. I’m pretty sure that if I tried something like that, they would push me away with a lame excuse||. When I was younger and in High School||, girls/*boys* avoided talking to me.|| Some of them kindly avoided me; they would say that they had something urgent to do||. Others were totally upfront instead.|| For example, one day, I was walking down the street|| and I got near to a good looking girl/*boy* that I had noticed at school.|| I just asked her/*his* name|| and she/*he* answered: “I am sorry, I am very busy and cannot waste my time!”|| I remember she/*he* was looking at me as if she/*he* was thinking: “I can’t even consider the idea of talking with you!”|| I felt very upset|| and frustrated|| and, as usual, I ran away and unburdened myself to my older sister|| Mary||.

It is difficult to describe in words how it feels to be rejected like that.|| It makes me feel like I am totally worthless||, like the skin of a banana on the corner of the street.|| But even that banana skin, I guess someone would care enough to throw it in the garbage. Me, not even that.|| Ok, now I am probably being too dramatic.|| It’s just that sometimes I can feel like such a nothing||.

In fact, my private life has been being very calm lately||, too calm||. I don’t have a girlfriend/*boyfriend*||. Women/*men* do not seem to be that into me||. I mean, I had two girlfriends/*boyfriends*|| but we split up badly||. My first girlfriend*/boyfriend*||, Ann/*David*,|| thought I was a bit apathetic||. She/*he* said she/*he* didn’t have fun with me||. We were together for 5 months|| but I could see from her/*his* face that she/*he* was never enthusiastic||. She/*he* didn’t talk to me about any relationship problems||, so I thought everything was normal.|| Then, one day, while wearing the yellow sweater|| I bought her/*him* for her/*his* birthday||, she/*he* said: “I am afraid you are not the man/*woman* for me”||. My latter girlfriend/*boyfriend*||, Lisa/*Simon*,|| she/*he* was super fun.|| I adored her/*him* at first||. After 3 months|| she/*he* dumped me|| for one of my best friends|| (not Simon/*Kate*, another one).|| Now, tell me that the banana skin metaphor is not appropriate||! But I’m optimistic,|| I think that I’ve just not found the right person yet.|| And I know that these things don’t come easy.|| Lots of people say that they found their true love in their 30s.|| I strongly think that to find Miss/*Mr* Right, you have to be mature|| and also lucky.||

My ideal girlfriend/*boyfriend* should be very sensitive||. She/*he* must be interested in what I do,|| and remember what is going on in my life.|| For example, Ann/*David*,|| my former girlfriend/*boyfriend* forgot about my birthday||. I didn’t want a big surprise or an amazing present, I just wanted to be told something||, or to receive a romantic birthday card||. But she/*he* completely forgot.|| This is what happened: she/*he* suggested to go out and spend a nice night together||. That night, she/*he* was holding a very big purse/*knapsack*,|| so I thought that maybe she/*he* put my gift inside it||, but at the end of the evening, we just went home|| and she/*he* didn’t even say “happy birthday”||. It made me feel very sad|| and disappointed||. I happen to forget appointments|| or assignments too,|| but I never forget very important events,|| especially when they involve the person I love.|| But then again, maybe Ann/*David* did not love me.|| Maybe Ann/*David* remembers important events too.|| My birthday was just not an important event to her/*him*||. So I decided to get in my car and start driving||, listening to the radio loudly|| and blaming myself for allowing someone to hurt me.||

During the weekend I like doing some physical activity.|| I do some jogging|| in Bellwoods Park|| on Saturday||, but I know that is not enough|| and I want to start going to the gym||. A few days ago|| I went to a gym|| on Bloor & Bathurst|| to ask for information.|| The place was amazing!|| It had more machines|| and instruments you could imagine||, and a great team of trainers||. One of those trainers got close to me and explained to me how they work with their clients||: their principal aim is to make them feel at home||. It made me feel very comfortable||. Anyway, everybody knows that working out with a friend is more fun than alone||, so I think I will ask my friend|| Patrick/*Lisa*|| to join me||. When I was younger||, I used to go to the swimming-pool||. I liked that so much||, but I had to quit|| because my spare time got very limited||: homework||, friends||, etc. Moreover, I had long hair at the time||, so it was very uncomfortable to dry it each time,|| it took so long.|| But now I am committed to start working out seriously||. I’ve heard it’s very healthy|| and cheers you up.||

I like watching movies||, in particular action movies;|| those ones have amazing special effects.|| One of my favorite movies is “The Day After Tomorrow.”|| Even though the story is a little trivial,|| I was impressed by the director’s ability to make everything appear so real!|| I remember that the first time I saw that movie I was with Patrick/*Lisa*.|| The theatre was really packed,|| but he/*she* managed to find two places|| right in the middle of the theatre||, the last two!|| The other people had to sit right in the front, destroying their neck||. I don’t like watching a movie if I am not seated properly.||

#### Adam/*Jean* (unlucky at work; *Toronto version*)

My name is Adam|| and I am 30 years old||. I was born in Toronto,|| where I live with my family.|| My family is composed of four people||: my parents,|| my brother and I||. I’m having some problems with them, lately.|| Actually, I don’t think I’ve ever been in a good relationship with my family,|| especially my parents.|| When I was in High school,|| they were very worried about me:|| I didn’t study enough,|| and I loved to skip school.|| I still remember that day when my teacher||, Mrs. Brown,|| called my parents|| because I skipped school:|| when I got home, my mom asked me: “So what have you been up to at school?”|| And I answered: “Oh, Mrs. Brown didn’t come this morning,|| so my classmates and I spent two hours chatting and stuff like that||.” Well, my parent punished me|| and I couldn’t go out for a very long week.||

Still I kept on skipping school occasionally||, and my friend and I|| used to go to the Islands instead||. It was fun!|| My friend had a house there||, a cute|| old|| little house|| painted in ochre|| with a hammock|| in the front||. We used to stay there||, safe||, talking about life||. It was a refuge for me.|| Truth is, I felt bad at school||. I had extreme difficulties concentrating||. For example, I could not stay concentrated on the lesson for more than 15 minutes.|| So I was often distracted,|| and I was never prepared||. I was one of the worst in my class||. Nobody believes me||, they all think I was lazy||, but I swear, I tried to study, but my head just flew somewhere else||. It was a frustrating feeling||. Like that day when I forgot my homework at home||: my teacher really believed that it was just one of my excuses||, but if I weren’t that distracted, I would have remembered to bring them at school.||

I have a girlfriend/*boyfriend*||, Julia/*Jack*,|| who lives here in Toronto.|| It’s a very serious relationship.|| I met her during a birthday party.|| I didn’t feel like going there,|| because I was tired:|| I had spent all night with my friends||. But I decided to go there anyway.|| It was on May|| 28||, 2007||, I won’t ever forget that day||. I was leaning against the wall||, thoughtfully drinking my “manhattan”|| and looking at people chatting and dancing||. Everybody looked like anyone else to me||. Suddenly, a girl/*boy* turned to me||: she/*he* had long black/*short blond* hair|| and was wearing a blue dress/*nice shirt*||, very simple||; I would say Armani style||, which fitted her/*him* so good||. Then we looked at each other and that was it: we’ve been together ever since.|| Now that I am older, I regretfully think that maybe, if I had done better at school, now I would have a good job|| and I could afford a house where I could live with Julia/*Jack*.|| We saw a place that would be perfect for us||. The rent is quite high:|| 1300$ per month, inclusive||. It’s a big apartment|| in Markham||. The owners are two Italians|| who live in the apartment upstairs.|| The house is made of red small bricks|| and it has a big back-yard with a lot of vegetables||: cucumbers,|| tomatoes,|| fennels||… and even three|| fruit trees||. The apartment is very bright.|| But even thinking about it is a waste of time||: I can’t afford any house at the moment|| and this makes me feel like a nobody.||

It will take a long time to get a good job||. Sometimes I think I won’t ever have success in the future,|| because I often fail||, and that is very hard to deal with||. I couldn’t even keep my job|| as shop assistant|| at Canada Tire||. Actually I didn’t like the job||: I was forced to wear that stupid|| white t-shirt|| with the name of the store on.|| I used to get confused|| because of all the stuff they were selling||. There were 1000 different types of plugs||, and lamps,|| and I used to forget some of them||. Then they fired me.|| Now I’m attending Physical Education at University|| and I like it||, but I am slower than my classmates||, and I just took two exams|| in two years||. A few months ago||, my classmates and I were walking on College Street,|| in Little Italy,|| enjoying a very nice|| and warm afternoon.|| Even though it was a relaxing afternoon||, my classmates couldn’t stop talking about exams and deadlines||. So, since I was getting very sick and tired||, I said: “Guys, why don’t we just enjoy this nice weather and set aside our worries?”||. And Paul/*Miranda*,|| a guy/*girl* I don’t like too much||, answered: “It’s very easy to say so when you missed most of your deadlines!||” I felt very uncomfortable|| and I thought I shouldn’t have talked at all||. Because of all these limitations, I am not positive about the house thing,|| and I feel guilty when I think about Julia/*Jack*.||

Once I made a plan||. I would get a job|| and try to make them notice me||. The point would be to become very indispensable||, so that they won’t be able to go on without me.|| At that point, they’ll pay me lots of money||. It’s not that simple, I know.|| I had been hired recently,|| but I had a hard time with my boss||. He kept on saying that I had mistakenly filed some invoices|| and that was a disaster.|| I tried to answer that it wasn’t my fault,|| because nobody had explained that to me,|| but he wasn’t listening.|| Then he said: “You have no idea how many guys/*girls* are willing to be hired in place of you!”|| I felt so completely useless||. Can you imagine how unnecessary I felt at that time?|| And I tried to think more positively, but it didn’t work.|| I still think I could do well in a job that does not require much attention and organization,|| but instead more imagination and creativity.|| For example, I love acting,|| and writing plays.|| I never leave home without my notebook||, so that I can write down hypothetical dialogues anytime I get the inspiration.|| Unfortunately, this kind of skill tends not to be valued.|| At least, my parents did not value them.||

Sometimes, in high school,|| I used to get good marks in acting class||. My parents used to say: “If you want to pursue this passion, at least you have to be the best in your class”||. I always answered to them: “If you truly believe in something, you can make it happen.”|| But they used to look at me like they couldn’t understand what I was saying.|| In these situations I usually feel so unappreciated.|| It’s really tough.|| They probably wanted to discourage me from engaging in a very difficult career||, they wanted me to stop dreaming||. But in fact, the message I got was that I was never good enough.|| And that doesn’t sound good at all.||

Besides that, my life goes on as usual.|| I hang out with my friends,|| I regularly go to the gym|| and I spend much time with my girlfriend/*boyfriend*.|| My friends and I would love to go to Ibiza|| next summer|| and we’re saving money for that.|| If we go there, it will be fun,|| like last summer in Greece.|| Julia/*Jack* was there too,|| so it was fun and romantic at the same time.|| We visited the most famous places there|| and I loved the Acropolis of Athens.|| It is very charming|| because you can picture the ancient Greeks walking around|| and going to the temples to pray.||

I also enjoy watching movies|| with my friends|| and our girlfriends/*boyfriends*,|| at my place,|| where I have a very big TV||. I/*Jack* usually choose the movies,|| because I/*he* know/*s* what my friends like.|| But, sometimes, I/*he* rent/*s* some horror movies on purpose||, because I like to get scared and jumpy/*and this really bothers me*.|| Obviously, we all know that most girls don’t like this kind of movie, because they/*we*’re chicks.|| So my friends and I/*the guys* make fun of them/*us*, when they/*we* scream during the scariest scenes.|| One night, Steven,|| one of our friends,|| turned the TV and the lights off,|| while we were watching “The Ring.”|| The girls/*the girls and I* started screaming.|| But among their/*our* screams, we could hear a masculine voice|| and we couldn’t figure out who it was.|| When I turned the light on,|| we found Steven,|| lying on the floor||, complaining,|| because he had hurt his ankle while fumbling in the dark.|| That time, the girls/*the girls and I* got their/*our* revenge,|| and the guys and I/*the guys* had one more reason to burst laughing.|| We were very mean,|| because we kept on making fun of him for a week||. But, the next night we spent together,|| Steven got his revenge||. He decided to buy popcorn for everyone||, because, as he told us, it was a very special event for him.|| So we started wondering that maybe he had found Miss Right, finally||. When we all gathered on the couch,|| I asked him: “Ok, tell us what’s going on”||. And he answered: “Well, it’s sad news||: I’m leaving the city tomorrow||, because I found a job in New York City.”|| I got very sad|| and upset|| because he hadn’t talked about that before||. So we quickly decided to have a party for him,|| to say goodbye.|| After that party||, we discovered that he made up this story to get revenge.|| I remember that, immediately after I realized that it was a joke||, I felt very mad at him||. But now I’m OK.||

#### Mike/*Susan* (unlucky in love; *Chicago version)*

My name is Mike/*Susan*|| and I am 25 years old.|| I was born in Dublin,|| but now I live in downtown Toronto,|| in my own flat||. The flat is on Queen West,|| on the second floor|| of a Victorian house|| that is painted in red|| and with a blue ceiling.|| I still remember the moment I decided to rent this flat.|| I was wandering in Queen West when I bumped into this enchanted house for rent.|| The landlord was there||, a funny|| Portuguese guy.|| We talked for a while and the deal was done!|| I am so happy to live on Queen West.|| Yet, I’m very close to my family|| and sometimes it’s tough to be apart||. I spent such a pleasant childhood|| in Dublin||, mainly because we lived on a farm.|| I can still smell the cookies|| that my granny used to bake|| on Sunday mornings.||

I have always known I would leave though||. When I was in sixth grade||, I got an award from my school|| for the best essay|| about organ donation||. I remember I was so proud of myself||. Even though I was really young, I was able to explain the pros and cons about organ donation.|| Since I was very young, I have always been interested in the field of health||.

After graduating in Pharmacy|| at the University of Toronto,|| I found a job in the Pharmaceutical industry||. The name of the industry is “Atom”||, it is located downtown, between Dundas and Manning.|| You might have noticed the place, it is a colored building|| and there is a dog at the entrance||, Zac||, a very cute beagle||. I am fairly happy with the job|| and I especially enjoy working with my colleagues.|| They are of my age,|| and collaborative||. I feel good there|| and it is important because, apart from them, I do not have many friends here in Toronto.||

I mean, I have a very small group of close friends||. We enjoy drinking beer while watching baseball games or planning trips/*We enjoy drinking coffee while watching TV or planning trips*||. Sometimes we go to our favorite club to hit on girls/*boys*||. Our favorite club is “Coca”||. It is on Queen West||, very close to my place.|| One of my closest friends is Simon/*Kate*.|| He’s a very good looking guy/*She’s very pretty*,|| and, when we all hang out together, he/*she* always gets the cutest girls/*boys*.|| Simon/*Kate* doesn’t need to try very hard to attract girls/*boys*||. He/*She* just starts talking about random topics and they listen to him/*her*.|| I’m pretty sure that if I tried something like that, they would push me away with a lame excuse||. When I was younger and in High School,|| girls/*boys* avoided talking to me.|| Some of them kindly avoided me; they would say that they had something urgent to do||. Others were totally upfront instead.|| For example, one day I got near to a good looking girl/*boy* that I had noticed at school.|| I just asked her/*his* name|| and she/*he* answered: “I am sorry, I am very busy and cannot waste my time!”|| I remember she/*he* was looking at me as if she/*he* was thinking: “I can’t even consider the idea of talking with you!”|| I felt very upset|| and frustrated|| and, as usual, I ran away and unburdened myself to my older sister|| Mary.||

It is difficult to describe in words how it feels to be rejected like that.|| It makes me feel like I am totally worthless||, like the skin of a banana on the corner of the street.|| But even that banana skin, I guess someone would care enough to throw it in the garbage. Me, not even that.|| OK, now I am probably being too dramatic here.|| It’s just that sometimes I can feel like such a nothing||.

In fact, my private life has been very calm lately||, too calm||. I don’t have a girlfriend/*boyfriend*||. Women/*Men* do not seem to be that into me||. I mean, I had two girlfriends/*boyfriends*,|| but we split up badly||. My first girlfriend/*boyfriend*||, Ann/*Sam*,|| thought I was a bit apathetic||. She/*He* said she/*he* didn’t have fun with me||. We were together for 5 months,|| but I could see from her/*his* face that she/*he* was never enthusiastic||. She/*He* didn’t talk to me about any relationship problems||, so I thought everything was normal.|| Then, one day, while wearing the yellow sweater|| I bought her/*him* for her/*his* birthday||, she/*he* said: “I am afraid you are not the man/*woman* for me”||. My latter girlfriend/*boyfriend*||, Lisa/*Ramon*,|| was super fun.|| I adored her/*him* at first||. After 3 months|| she/*he* dumped me|| for one of my best friends|| (not Simon/*Kate*, another one).|| Now, tell me that the banana skin metaphor is not appropriate||! But I’m optimistic,|| I think that I’ve just not found the right person yet.||

My ideal girlfriend/*boyfriend* should be very sensitive||. She/*He* must be interested in what I do,|| and remember what is going on in my life.|| For example, Ann/*Sam*||, my former girlfriend/*boyfriend*, forgot about my birthday||. I didn’t want a big surprise or an amazing present, I just wanted to be told something||, or to receive a romantic birthday card||. But she/*he* completely forgot.|| This is what happened: She/*he* suggested to go out and spend a nice night together||. That night, she/*he* was holding a very big purse/*knapsack*,|| so I thought that maybe she/*he* put my gift inside it||, but at the end of the evening, we just went home|| and she/*he* didn’t say even “happy birthday”||. It made me feel very sad|| and disappointed||. I happen to forget appointments too,|| but I never forget very important events,|| especially when they involve the person I love.|| I don’t like to say it,|| but maybe Ann/*Sam* did not love me.|| Maybe Ann/*Sam* remembers important events too.|| My birthday was just not an important event to her/*him*||. So that night I decided to get in my car and start driving||, listening to the radio loudly|| and blaming myself for allowing someone to hurt me.||

During the weekend I usually do some physical activity.|| A few days ago|| I went to a gym|| on Bloor & Bathurst|| to ask for information.|| The place was amazing!|| Also it has a great team of trainers||. One of those trainers,|| Ricky||, a very tall guy|| got close to me and explained to me how they work with their clients||: their principal aim is to make them feel at home|| and then they start asking you some questions|| in order to get to know you better||, because they really want to create the right working out schedule for your health||. It made me feel very comfortable||. When I was younger||, I used to go to the swimming-pool||, I loved it||, but I had to quit|| because my spare time got very limited.|| But now I am committed to start working out seriously||. I’ve heard it’s very healthy|| and cheers you up.|| Rickie, the trainer,|| told me that people usually take nine months|| to see the first improvements,|| in terms of losing weight and becoming more tonic.||

I like watching movies||, in particular action movies||. One of my favorite movies is “The Day After Tomorrow.”|| Even though the story is a little trivial,|| I think that the director was successfully able to make everything appear so real!|| I remember that the first time I saw that movie I was with my friend Patrick/*Kate*.|| The theatre was really packed,|| I truly wanted to leave and to come back another day||, but then Patrick/*Kate* managed to find two places|| right in the middle of the theatre||, the last two!|| The other people had to sit right in the front, destroying their neck||.

#### Adam/*Jean* (unlucky at work; *Chicago version)*

My name is Adam/Jean|| and I am 25 years old||. I was born in Toronto,|| where I live with my family.|| My family is composed of four people||: my parents,|| my brother and I||. I am really fond of my brother,|| Trevor,|| even though we sometimes fight||; it happens when you have to share your room with someone else.||

I think that my life hasn’t always been easy||. For example, when I was at school, I immediately realized I could not stay concentrated on the lesson for more than 15 minutes.|| So I was often distracted,|| and never prepared||. I was one of the worst in my class||. My teachers didn’t believe me||, they all thought I was lazy||, but I swear, I tried to study, but my head just flew somewhere else||. It was a frustrating feeling||. I remember the good students chatting with professors about topics of mutual interest, or even just about the lesson||. I would have loved to be able to feel part of that group||. But I just was not good enough for that,|| and I have always felt estranged because of this.||

In order to escape from those frustrating feelings of incompetence,|| I used to skip classes, as many students do||. I always smile by myself|| when I remember that day when my teacher,|| Mrs. Brown,|| called my parents|| because I skipped school.|| When I got home, my mom asked me: “So what have you been up to at school?”|| And I answered: “Oh, Mrs. Brown didn’t come this morning,|| so my classmates and I spent two hours chatting and stuff like that||.” Well, my parent punished me|| and I couldn’t go out for a very long week.|| I remember I got very upset.|| I even remember what I did instead of going to school, that time: I went to the Islands||. It was fun!|| My friend had a house there||, a cute|| old|| little house,|| painted in ochre,|| with a hammock|| in the front||. We used to stay there||, safe||, talking about life||. It was a refuge for me.||

I am afraid it will take a long time for me to get a good job||. Sometimes I think I won’t ever have success,|| because, let’s face it, I often fail workwise||, and that is very hard to deal with||, because work is an important part of our lives.|| For example, I couldn’t keep my job|| as shop assistant|| at Canada Tire||. The most exhausting thing about that job was that there were selling 1000 different types of plugs||, and lamps,|| and I used to forget some of them||. Then they fired me.|| At the moment I’m attending Physical Education at University|| and I like it||, but I am slower than my classmates||, and just took two exams|| in two years||. A few months ago||, my classmates and I were walking on College Street,|| in Little Italy,|| enjoying a warm afternoon.|| Even though it was a relaxing afternoon||, my classmates couldn’t stop talking about exams and deadlines||. So, since I was getting very sick and tired|| – and defensive, I confess-||, I said: “Guys, why don’t we just enjoy this nice weather and set aside our worries for a bit?”||. And Paul,|| a guy I don’t even like||, answered: “It’s very easy to say so when you missed most of your deadlines||.” I felt very uncomfortable|| and thought I shouldn’t have talked at all||.

Once I made a plan||. I would get a job|| and try to make them notice me.|| It’s not that simple, I know.|| I have been hired recently,|| but I already had a hard time with my boss||. He said that I had mistakenly filed some invoices|| and that was a disaster||. Then he said: “You have no idea how many guys/*girls* are willing to be hired in place of you!”|| I felt so completely useless||. Can you imagine how unnecessary I felt at that time? || I keep thinking that if I had done better at school and gotten a good job,|| now I could afford a house to live by myself.|| I saw a place that would be perfect for me, the other day||. I liked it very much.|| The rent is quite high:|| 1300$ per month, inclusive||. It’s a big apartment|| in Markham||. The owners are two Italians|| who live in the apartment upstairs.|| The house is made of red small bricks|| and it has a big back-yard with a lot of vegetables||: cucumbers,|| tomatoes,|| fennels||… and even three|| fruit trees||. The apartment is very bright.|| But I can’t afford any house at the moment because of all my limitations,|| and this makes me feel like a nobody.|| And I feel guilty when I think about it:|| I think the reason I don’t have a house is that I am not good enough.|| Sometimes I am more positive though,|| and think I could do well in a job that does not require much attention and organization,|| but instead more imagination and creativity.|| For example, I love acting||, and writing plays.|| I never leave home without my notebook||, so that I can write down hypothetical dialogues anytime I get the inspiration.|| Unfortunately, this kind of skill tends not to be valued.|| I took part in many competitions,|| during High School.|| They were a chance for me to show how good I was in acting||. When I didn’t win, I used to feel terrible,|| because I had to see the arrogant look of the winner||, that seemed to say: “You won’t be able to do better.|| You’re a loser.”|| And it’s really tough,|| because it makes me feel like I don’t have any chance to get better in the future.|| I would stay awake all night in those days.||

Besides that, my life goes OK.|| I hang out with my friends|| and I regularly go to the gym.|| My friends and I would love to go to Ibiza|| next summer|| and we’re saving money for that.|| If we go there, it will be fun,|| like last summer in Greece: it was fun.|| We visited the most famous places there and|| I loved the Acropolis of Athens.|| It is very charming,|| because you can picture the ancient Greeks walking around|| and going to the temples to pray.|| I also enjoy watching movies|| with my friends,|| at my place||, where I have a very big TV||. I usually choose the movies,|| because I know what my friends like.|| But, sometimes, I rent some horror movies on purpose||, because I like to get scared and jumpy.|| Most girls don’t like this kind of movies||, so my friends and I make fun of them, when they scream during the scariest scenes.|| One night, Steven,|| one of our friends,|| turned the TV and the light off,|| while we were watching “The Ring.”|| The girls started screaming.|| But among their screams, we could hear a masculine voice|| and we couldn’t figure out who it was.|| When I turned the lights back on,|| we found Steven,|| lying on the floor|| complaining,|| because he had hurt his ankle while fumbling in the dark.|| I laughed so much! || That time, the girls/*the girls and I* got their/*our* revenge,|| and the guys and I/*the guys* had one more reason to burst out of laughing.|| We kept on making fun of him for a whole week||.

### Faux pas scenarios

#### Love scenarios

1. “Tonight we’ll go to the restaurant!” said [VICTIM] to his/*her* partner, who was singing in the shower. He/*She* had planned to have a nice evening with her/*him* because it was their anniversary. While he/*she* was secretly packing the present for her/*him*, he/*she* wondered whether his girlfriend/*her boyfriend* also remembered their anniversary. Then she/*he* came out of the shower. “What were you saying honey? I couldn’t hear from the shower,” asked the girl/*the boy*. [VICTIM] said: “I thought it would be nice to go out for dinner since today…” But she/*he* interrupted him/*her*: “Oh sorry honey, not today: I promised some colleagues I would join them for a drink! What about next Saturday?”
2. [VICTIM] was in Lisbon for a conference. He/*She* had met a lot of colleagues from abroad, who were working on similar topics as he/*she* was, but his favorite colleague was Valentina/*Luca*, an extroverted lawyer from Italy. On the last night in Lisbon, [VICTIM] and Valentina/*Luca* went for dinner together. After dinner, they took a nice walk through Bairro Alto. [VICTIM] said: “Lisbon is such a charming city.” Valentina*/Luca* replied: “Yes, I agree, even though I think that visiting it with your partner would be a completely different story!” [VICTIM] replied: “I see.”
3. [VICTIM] had shopped around all day and was very tired. He/*She* was looking for a seat, when he/*she* saw a bench in a park, with a very cute girl/*boy* sitting there. [VICTIM] thought that it would be great to sit by her/*him*. He/*She* got closer to the girl/*boy* and asked: Hi, do you mind if I have a seat here, or are you waiting for someone? The girl/*boy* replied: “No problem, I’m not waiting for anyone but, as you can see, the park is not too busy and there are a lot of empty seats.”
4. [VICTIM] and his girlfriend/*boyfriend* met at a bar on the beach. They decided to take a walk close to the sea. [VICTIM] thought everything was very romantic: the sound of the waves, the moonlight, and the noise of the music that was now far away… “Hey,” said [VICTIM], “Let’s buy some red wine and come back here!” His girlfriend/*boyfriend* smiled. [VICTIM] asked: “Why are you smiling?” She/*He* replied: “Nothing important… It’s that this place reminds me of my last boyfriend/girlfriend; we had such a great time last summer on this beach.”
5. [VICTIM] was having problems with his new girlfriend/boyfriend. He was drinking a hot chocolate at a Café, thinking about his/her problems, when Peter, a guy he/she barely knew, came over and said: “Hi [VICTIM], how are you? You look so sad… Don’t tell me it’s a trivial love issue! Love is so overrated these days!” [VICTIM] answered: “Well, I guess the situation is more complicated than that…”
6. [VICTIM] and his girlfriend/*her boyfriend* went for an ice cream on College Street. [VICTIM]’s girlfriend/*boyfriend* had a double chocolate ice cream, whereas [VICTIM] tried the new blue flavor. His/*her* mouth went blue in less than 2 minutes. [VICTIM]’s girlfriend/*boyfriend*, looking for a Kleenex in her purse/*his bag*, took out a CD instead. “Hey, what is this CD?” asked [VICTIM]. “Oh nothing special, it’s a compilation of my favorite love songs, answered [VICTIM]’s girlfriend/*boyfriend*. “Oh thank you!!! Finally, you made me a CD!” exclaimed [VICTIM]. “Actually, I made this for my friend from the acting school. Did I ever tell you about him/*her*?”
7. [VICTIM] and his girlfriend/*her boyfriend* had been together for 2 weeks. However, [VICTIM] had to fly to Europe for a holiday that he/*she* had planned before meeting her/*him*. During his/*her* stay in Paris, [VICTIM] thought a lot about his girlfriend/*her boyfriend*. He/*She* decided to buy her/*him* a little present. He/*She* bought a little depiction of the Eiffel Tower made by a street painter. The painting included a couple kissing each other. [VICTIM] thought it was so romantic. At the airport, [VICTIM] and his girlfriend/*her boyfriend* gave each other a huge hug. “Did you miss me?” asked his girlfriend/*her boyfriend*. “Oh, I missed you so much. I got you a present,” replied [VICTIM]. She/*he* said: “That’s so nice! I bet it’s something high-tech and not some crappy souvenir.”
8. [VICTIM] was talking on the phone with his girlfriend/*her boyfriend*. He/*she* was telling her/*him* about his/*her* frantic morning, and some problems he/*she* had at work with his PhD supervisor. “… and after I waited to talk to him for the entire day…” [VICTIM] said, “…guess what he told me? He told me to meet the next week! Can you believe that? I wasted so much time waiting!” His girlfriend/*her boyfriend* did not answer. “Hey, did you hear what I said?” [VICTIM] asked.” Sure, sure,” she/*he* answered, “it’s just that I was trying to send an email, but it keeps bouncing back.” “I see” said [VICTIM], “did you double-check the address?”

#### Work scenarios

1. [VICTIM] went to say bye to the director of his/*her* office, because he/*she* was planning to leave for the holidays. “Hi Dr. Henson,” he/*she* said, “I wanted to say bye. I am heading to Italy. Should you need to contact me, please don’t hesitate to do so by email.” “Don’t worry,” answered the director “I don’t think your absence will cause huge problems. Have fun!”
2. [VICTIM] was about to graduate. The same day that he/*she* was informed his/*her* thesis had been accepted by the committee, he/*she* met an old friend from high school, Antonio, with his new girlfriend. “Hi [VICTIM], how are you doing?” asked Antonio. “I am doing great! I just found out that I am going to graduate in a few weeks!” said [VICTIM]. “Wow,” said Antonio, congratulations! By the way, let me introduce you my girlfriend, Tiffany. “Oh hi Tiffany, how are you doing?” asked [VICTIM]. Tiffany replied, “Not very well, actually. I am very busy. I am graduating too, but in Physics, which, as you can imagine, is among the most difficult studies…”
3. [VICTIM] was taking a walk, when he ran into his/*her* former professor of Psychology. “So, [VICTIM], did you finally manage to get a job?” the professor asked. “Yes, I found a job as a shop assistant on Queen street west. I was so happy when they hired me,” he/*she* said. “How is that going?” asked the professor. “Oh it’s not bad,” said [VICTIM], casually.
4. [VICTIM] was very happy because he/*she* had obtained a B- in his/*her* favorite subject. Since he/*she* had been taking lessons for only 2 years, he/*she* was proud of his/*her* grade. He/*She* immediately called his*/her* father to tell him about the great news. “Dad,” he/she said, “You know what happened? The instructor finally gave us the grades for the projects!” His/*Her* father exclaimed: “Oh my God! Are you trying to say you got an A???” [VICTIM] replied: “Oh, no, just a B-, but I will do better next time.”
5. [VICTIM] was standing by the cashier in a restaurant, waiting to pay. A guy, seated at a table near [VICTIM], accidentally spilt some coffee on the floor. “I’ll get you another cup of coffee,” said the waiter to the guy. After a while nobody had come to clean the spilt coffee, so the guy went up to [VICTIM] and said, “I spilt some coffee over by my table. Could you please mop it up?”
6. [VICTIM] had taken a computer course for adults. He/*She* had studied lots of mathematics at school, and he/*she* thought he/*she* was one of the best at the course. He/*she* would have loved to get the highest grade in the final evaluation. The day the grades were made available, he/*she* found out that he/*she* only got B, whereas another guy from the class got A-. He/*Sh*e, therefore, decided to go ask the professor what he/*she* did wrong. As soon as the professor saw him/*her*, he said, “Congratulation [VICTIM]! It is such a great grade for you, eh? You must be very happy!”
7. [VICTIM] was sick and tired of his/*he*r current job. He/*She* needed something more challenging. He/*She* decided to make a change to his/*her* life and ask for a promotion to his/*her* boss. He/*She* told his mother about this decision. His/*her* mother was a very adventurous woman and she would have understood. But instead she appeared strangely cold. “What do you think, mom?” [VICTIM] asked. She said: “I was just thinking… are you sure that afterwards your new assignments won’t be too difficult for you? Nevermind, you still have some time to think about it…”
8. [VICTIM] At Fernhaven school, there was a writing competition. Everyone was invited to enter. [VICTIM] loved the story he/*she* had entered in the competition. A few days later, the results were announced: [VICTIM]’s story had not won anything and a classmate, Jake, had won the first prize. The following day, [VICTIM] was talking to Jake. Jake said, “It was so easy to win that contest. All of the other stories in the competition were boring.” “Where are you going to put your trophy?” asked [VICTIM].
9. [VICTIM] had been hired in a Canadian company. One day, he/*she* presented his/*her* first project to 50 people, including his/*her* boss. The presentation was very interesting and well-done. After that, [VICTIM] got closer to his/*her* boss and asked him: “What do you think about it?” His/*Her* boss answered: “I think that you look very good with this tie/*shirt*. Is it new?”

#### Generic scenarios

1. [VICTIM] had just started with his/*her* new job on Bay Street. He/*She* decided to join his/*her* new colleagues for a dinner, in order to get to know them better. He/*She* was drinking a cocktail when a man/*woman* asked: “You’re the guy/*girl* who’s just transferred here, aren’t you? My name is Bob/*Sally*, nice to meet you.” Yes, replied [VICTIM], “Actually I started at this office last week.” “Last week?! That’s really weird! I thought you just arrived. Nobody has noticed you yet…” said Bob/*Sally*. “Oh, I see,” said [VICTIM].
2. [VICTIM]’s sister was throwing a surprise party for his/her birthday. She invited Sarah, a friend of [VICTIM]’s, and said: “Don’t tell anyone, especially [VICTIM].” The day before the party, [VICTIM] was over at Sarah’s and Sarah spilled some coffee on a new dress that was hanging over her chair. “Oh no!” said Sarah, “I was going to wear this at your party!” “What party?” said [VICTIM]. “Come on,” said Sarah, “Let’s go see if we can get the stain out.”
3. [VICTIM] and Jennifer were attending the same Math class at College. One day, they took a walk on the sea-front. “I love hip-hop music so much,” said [VICTIM]. “On Saturday nights I can’t wait to hang out with my friends to dance in clubs.” Jennifer said: “Oh, that’s very interesting. I like reading in my spare time, instead. These days I’m reading a book by Oliver Sacks.” “Who? Sorry, I didn’t hear you,” said [VICTIM]. Jennifer answered: “Never mind, I don’t think you know him.”
4. [VICTIM], during a break at work, asked his/*her* colleagues to come closer to him/*her*. “I have something to tell you,” he/*she* said. “John Morehouse, one of our accountants, is very sick with cancer and he’s in the hospital.” Everyone was quiet, absorbing the news, when Robert arrived late. “Hey, I heard this great joke last night!” Robert said. “What did the terminally ill patient say to his doctor?” [VICTIM] said: “Okay, let’s get back to work.”
5. [VICTIM] had just met an Italian colleague, Fabio/*Paola*, who had joined their work group. [VICTIM] wanted to invite Fabio/*Paola* for dinner to get to know him/*her* better. He/*She* asked Fabio/*Paola*: “Hey Fabio/*Paola*, why don’t you come over for dinner some time, we just bought a pasta machine and you could help us figure how to use it!” Fabio/*Paola* answered: “Oh why not… but I guess you should be able to get it to work. Was there an instruction manual?”
6. [VICTIM] bought his/*her* friend, Anne, a crystal bowl for a wedding gift. Anne had a big wedding and there were a lot of presents to keep track of. About a year later, [VICTIM] was over at Anne’s for dinner. [VICTIM] accidentally dropped a wine bottle on the crystal bowl and the bowl shattered. I’m really sorry. I’ve broken the bowl, said [VICTIM]. “Don’t worry,” said Anne. “I never liked it anyway. Someone gave it to me for my wedding.”
7. It was 9 p.m. and [VICTIM] was still doing his/*her* homework, because he/*she* had been watching TV all day long. He/*She* wasn’t that good in Math and he/*she* couldn’t go on with his/*her* homework. So he/*she* thought that his/*her* best friend could help him/*her*, and decided to call him/*her*, hoping he/*she* wouldn’t mind. “Hi, it’s [VICTIM], am I disturbing you?” asked [VICTIM]. “No, I really love being interrupted while I am watching my favorite TV program…” his/*her* friend replied.
8. [VICTIM] had just moved into a new apartment. [VICTIM] went shopping and bought some new curtains for his/*her* bedroom. When he/*she* had just finished decorating the apartment, his/*her* best friend came over. [VICTIM] gave her a tour of the apartment and asked, “How do you like my bedroom?” “Great, but those curtains are horrible,” his/*her* best friend said. “I hope you’re going to get some new ones!”
9. [VICTIM] had just started with his/*her* new job. One day, in the coffee room, he/*she* was talking to a new colleague, Andrew. “What does your father do?” Andrew asked. “He’s a lawyer,” answered [VICTIM]. A few minutes later, Claire came into the coffee room looking irritated. I just had the worst phone call ever, she said “Lawyers are all so arrogant and greedy. I can’t stand them.” “Do you want to come look over these reports?” Andrew asked Claire. “Not now,” she replied, “I need my coffee.”
10. [VICTIM] had won a first class flight to Las Vegas. He/*She* was enjoying his/*her* comfortable flight, when he/*she* heard two hostesses chatting in a very low voice: “Yes, I tried that lipstick too! But I think that it’s not as smudge-proof as they told in the advertisement on TV. After drinking, it gets the glass dirty,” said one of them. “Really?” answered the other, “Mine works perfectly. I think that something is wrong with yours.” “Excuse me,” said [VICTIM], “Can I have a glass of lemonade, please?” “Sure. But, I’m afraid that you’ll have to wait a little bit because we’re all busy at this moment,” answered one of the hostesses.

#### Neutral scenarios

1. [VICTIM] met his neighbor Peter/*Joan*, who was taking his dog Zack out to the park. Peter*/Joan* had just thrown a stick for Zack to chase. Peter*/Joan* and [VICTIM] chatted for a few minutes and, after a while, [VICTIM] asked: “Are you heading home? Would you like to walk together?” “Sure,” Peter*/Joan* said. He called Zack, but he was busy chasing pigeons and didn’t come. “It looks like he’s not ready to go,” he/*she* said. “I think we’ll stay.” “OK,” [VICTIM] said. “I’ll see you later.”
2. [VICTIM] went to the barber for a haircut. “How would you like it cut?” the barber asked. “I’d like the same style as I have now, only take about an inch off,” [VICTIM] replied. The barber cut it a little uneven in the front, so he had to cut it shorter to even it out. “I’m afraid it’s a bit shorter than you asked for,” said the barber. “Oh well,” [VICTIM] said, “it’ll grow out.”
3. [VICTIM] had had a major role in last year’s school play and he/*she* really wanted the lead role this year. He/*She* took acting classes all year, and in the spring he/*she* auditioned for the play. The day the decisions were posted, he/*she* went before class to check the list of who had made the play. He/She hadn’t made the lead but was cast in a minor role. He ran into his/*her* best friend in the hall and told him what had happened. “I’m sorry,” he said. “You must be disappointed.” “Yes,” [VICTIM] answered, “I have to decide whether to take this role.”
4. [VICTIM] was shopping for a shirt to match his/*her* suit. The salesman showed him/*her* several shirts. [VICTIM] looked at them and finally found one that was the right color. But when he/*she* went to the dressing room and tried it on, it didn’t fit. “I’m afraid it’s too small,” he/*she* said to the salesman. Not to worry, the salesman said. “We’ll get some in next week in a larger size.” “Great. I’ll just come back then,” [VICTIM] said.
5. [VICTIM] stopped off at the gas station on the way home to fill up his/*her* car. He/*She* gave the cashier his/*her* credit card. The cashier ran it through the machine at the counter. “I’m sorry,” said the cashier, “the machine won’t accept your card.” Hmmm, that’s funny, [VICTIM] said. “Well, I’ll just pay in cash.” [VICTIM] gave the cashier 20 dollars and said, “I filled up the tank with regular gasoline.”
6. [VICTIM] bought a new car, a red Peugeot. A few weeks later, he/*she* backed it into his/*her* neighbor Ted’s car, an old beat-up Volvo. His/*her* new car wasn’t damaged at all and he/*she* didn’t do much damage to Ted’s car either: just a scratch in the paint above the wheel. Still, he/*she* went up and knocked on the door. When Ted answered, [VICTIM] said, “I’m really sorry. I’ve just put a small scratch on your car.” Ted looked at it and said: “Don’t worry. It was only an accident.”
7. [VICTIM] was at the library. He/*She* found the book he/*she* wanted about hiking in the Grand Canyon and went up to the front counter to check it out. When he/*she* looked in his/*her* wallet, he/*she* discovered he/*she* had left his/*her* library card at home. “I’m sorry,” [VICTIM] said to the woman behind the counter. I seem to have left my library card at home.” “That’s OK,” she answered. “Tell me your name, and if we have you in the computer, you can check out the book just by showing me your driver’s license.”
8. [VICTIM] went to the butcher to buy some meat. It was crowded and noisy in the shop. He/*She* asked the butcher: “Do you have any free-range chickens?” He nodded and started to wrap up a roasted chicken for him. “Excuse me,” [VICTIM] said, “I must not have spoken clearly. I asked if you had any free-range chickens.” “Oh, sorry,” the butcher said, “we’re all out of them.”
9. [VICTIM] was at a party at his/*her* friend Oliver’s house. He/*She* was talking to Oliver when a woman came up to them. She was one of Oliver’s neighbors. The woman said hello to Oliver then turned to [VICTIM] and said, “I don’t think we’ve met. I’m Maria, what’s your name?” “I’m [VICTIM].” “Would anyone like something to drink?” Oliver asked.
10. [VICTIM] was waiting at the bus stop. The bus was late and an old lady had been standing there a long time. She looked very tired. When the bus finally came, it was crowded and there were not many seats left. [VICTIM] managed to get a seat. The old lady saw a neighbor, standing in the aisle of the bus. “Hello, Ma’am,” he said. “Were you waiting there long?” “About 20 minutes,” the old lady replied. [VICTIM] got up and said: “Ma’am, would you like my seat?”
